# Artificial Intelligence and Extended Reality in the Training of Vascular Surgeons: A Narrative Review

**DOI:** 10.3390/medsci13030126

**Published:** 2025-08-12

**Authors:** Joanna Halman, Sonia Tencer, Mariusz Siemiński

**Affiliations:** 1 Vascular Surgery Department, Medical University of Gdańsk, University Clinical Centre in Gdańsk, 80-952 Gdańsk, Poland; 2 Scientific Circle of Neurotraumatology, Department of Emergency Medicine, Medical University of Gdańsk, 80-210 Gdańsk, Poland; sonia.tencer@gmail.com; 3 Department of Emergency Medicine, Medical University of Gdańsk, University Clinical Centre in Gdańsk, 80-952 Gdańsk, Poland; sieminski@gumed.edu.pl

**Keywords:** virtual reality, artificial intelligence, vascular surgery training, simulation-based education, extended reality, patient-specific rehearsal, surgical education, endovascular simulation

## Abstract

Background: The rapid shift from open to endovascular techniques in vascular surgery has significantly decreased trainee exposure to high-stakes open procedures. Simulation-based training, especially that incorporating virtual reality (VR) and artificial intelligence (AI), provides a promising way to bridge this skill gap. Objective: This narrative review aims to assess the current evidence on the integration of extended reality (XR) and AI into vascular surgeon training, focusing on technical skill development, performance evaluation, and educational results. Methods: We reviewed the literature on AI- and XR-enhanced surgical education across various specialties, focusing on validated cognitive learning theories, simulation methods, and procedure-specific training. This review covered studies on general, neurosurgical, orthopedic, and vascular procedures, along with recent systematic reviews and consensus statements. Results: VR-based training speeds up skill learning, reduces procedural mistakes, and enhances both technical and non-technical skills. AI-powered platforms provide real-time feedback, performance benchmarking, and objective skill evaluations. In vascular surgery, high-fidelity simulations have proven effective for training in carotid artery stenting, EVAR, rAAA management, and peripheral interventions. Patient-specific rehearsal, haptic feedback, and mixed-reality tools further improve realism and readiness. However, challenges like cost, data security, algorithmic bias, and the absence of long-term outcome data remain. Conclusions: XR and AI technologies are transforming vascular surgical education by providing scalable, evidence-based alternatives to traditional training methods. Future integration into curricula should focus on ethical use, thorough validation, and alignment with cognitive learning frameworks. A structured approach that combines VR, simulation, cadaver labs, and supervised practice may be the safest and most effective way to train the next generation of vascular surgeons.

## 1. Introduction

Artificial intelligence (AI)-assisted virtual reality (VR) simulation has emerged as a promising tool for learning surgical skills, showing significant potential to enhance surgical performance in real clinical settings. The need for structured, simulation-based training is especially urgent in vascular surgery, a specialty undergoing rapid transformation with the widespread shift from traditional open procedures to minimally invasive endovascular techniques [1,2,3]. Although this paradigm shift has helped to improve patient outcomes and increase safety, it has also resulted in a substantial reduction in trainee exposure to open procedures. Proficiency in complex open procedures is essential, particularly in emergent or salvage scenarios. Examples include the explantation of infected stent grafts, the repair of ruptured aneurysms that cannot be treated by endovascular procedures, and major arterial reconstructions in younger patients [4]. Such technically demanding operations require adequate training in open surgical techniques to ensure the preparation of future vascular surgeons. VR simulation provides a unique opportunity to bridge this training gap by allowing residents to develop these critical skills in a safe, controlled, and risk-free environment outside the operating theater [5]. Current evidence confirms that VR-trained residents perform procedures more quickly and accurately than control groups [6,7]. VR training has been found to reduce procedural errors by 32–42% and improve the speed of skill acquisition, multitasking ability, and hand–eye coordination [8,9]. AI has also shown potential in assessing endovascular surgical competence and clinical decision-making [10]. In addition to their educational value, VR simulation platforms offer a significant economic advantage over traditional live animal training methods. A cost analysis by Berry et al. has shown that training with a VR simulator is substantially less expensive than using an animal laboratory. The cost per trainee was estimated at USD 3434 compared to USD 4634 when using animal models. Notably, if the simulator is rented rather than purchased, this cost drops even further to USD 1076 per participant, leading to potential savings of up to USD 1 million over five years [11]. Beyond cost-effectiveness, VR-based training has also shown comparable educational outcomes. In a related study, novices trained on VR simulators demonstrated similar improvements in their procedural skills in both the virtual environment and animal laboratories, suggesting the transferability of skills [12]. The aim of this review is to summarize and critically evaluate the current evidence on the role of AI-integrated VR training in enhancing the technical skills and improving the surgical performance of vascular surgery trainees. To identify the relevant literature, we conducted a narrative review using the PubMed database, focusing on studies published in recent years. Search terms included combinations of “virtual reality”, “extended reality”, “augmented reality”, “mixed reality”, “simulation-based training”, “surgical education”, “vascular surgery”, “artificial intelligence”, and “machine learning”. Articles were included if they discussed AI or XR applications in surgical education, particularly those focused on technical skill development, cognitive learning theories, or training program design. We considered original research articles, systematic reviews, and consensus statements published in English with full-text availability. While the primary focus was vascular surgery, we included representative studies from other surgical specialties to provide broader context and support cross-disciplinary comparisons.

## 2. Terminology

Extended reality (XR) is a broad term that covers the entire spectrum of immersive experiences, from fully virtual environments to digital overlays of the real world.

VR refers to a fully computer-generated environment in which users can interact with a simulated scenario. VR is particularly suitable for medical education and surgical training. It enables safe, repeatable practice of procedures, simulation of complex cases, and detailed anatomical exploration in a controlled environment [13,14,15].

Augmented reality (AR) adds digital features such as 3D anatomy, instructions, or images to what the user sees in the real world. Unlike VR, which blocks out the real world entirely, AR allows users to stay grounded in their physical surroundings while viewing helpful virtual content overlaid on top. In the surgical setting, AR contributes meaningfully to both planning and execution by superimposing individualized anatomical information onto the live surgical field. This enables the operating team to visualize structures not visible on the surface, thereby improving precision in high-risk procedures such as vascular reconstructions or oncologic resections [16].

Mixed reality (MR) combines virtual content with the real world in a dynamic and interactive way. Unlike AR, which simply overlays digital elements onto the physical environment, MR enables real-time interaction between virtual objects and the physical space. This means that 3D digital content, such as anatomical models or surgical guides, is not only visible but also anchored in space and responsive to user movements, gestures, and environmental changes. In surgical settings, MR allows clinicians to work with patient-specific holograms, such as vascular reconstructions or organ models, directly within the operating room. These holograms can be rotated, zoomed, or virtually dissected, enhancing anatomical visualization, spatial awareness, and procedural planning. Additionally, MR platforms facilitate remote collaboration, enabling experts in different locations to view and interact with the same holographic content simultaneously in real time, which is especially useful in complex or rare cases [17].

Artificial intelligence (AI) and its subset, machine learning (ML), are computational methods that analyze large datasets, recognize complex patterns, and facilitate data-driven decisions with minimal human input. In medical education and clinical practice, AI and ML are increasingly used to monitor, improve, and customize training and treatment. In surgical training, AI-powered systems can assess procedural performance, identify errors, and provide adaptive, real-time feedback based on the learner’s skill level. These tools allow for objective skill evaluation and aid in creating personalized learning paths. Clinically, AI and ML are employed in medical image analysis, risk assessment, and predicting outcomes, helping clinicians make faster, more accurate decisions. Incorporating these technologies into training and patient care enhances healthcare efficiency, precision, and personalization [18,19,20,21].

## 3. Cognitive Theories: The Scientific Basis for Why VR Training Works Effectively

**Figure 1:**
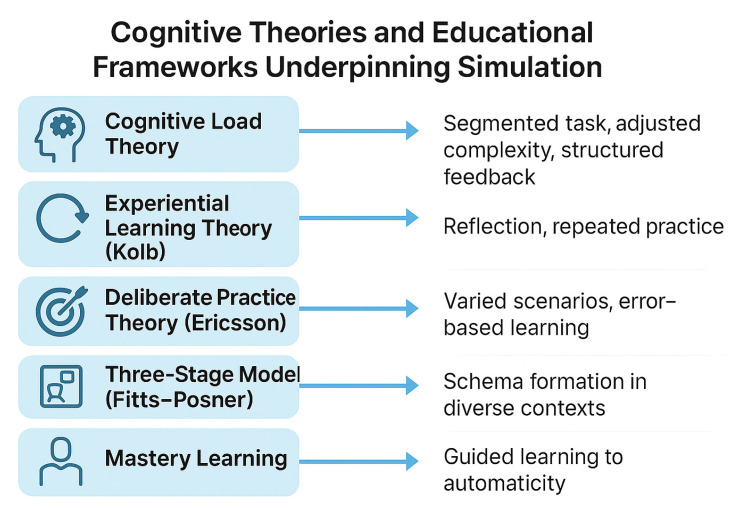
Cognitive theories and educational frameworks supporting simulation.

Surgical simulation is most effective when it is based on how people learn. This paragraph reviews key learning theories. Kolb’s experiential learning cycle explains that we learn through action, reflection, understanding, and repetition. This aligns well with VR simulation, which encourages repeated, hands-on practice and reflection. Another theory, called situated learning, suggests that people learn more effectively when the training is realistic, such as when VR uses patient-specific cases. However, learning can become more difficult if the brain becomes overloaded. Cognitive load theory reminds us that beginners have limited mental capacity, so it is better to keep things simple and avoid too much detail early on. Skills evolve gradually: initially, we think consciously, then become more fluid, and finally perform actions automatically without needing to think. This progression is explained by the Fitts–Posner model. Practice helps, but it works best when it is goal-oriented and includes feedback. This is known as deliberate practice. Schema theory suggests that modifying training enables learners to apply their skills in various situations. Mastery learning shows that everyone can succeed given enough time, feedback, and support. VR is ideal for this, as it enables unlimited, safe practice. AI can further improve training by giving real-time, personalized feedback. Together, these ideas help design smarter, more effective VR training for surgery [7,15,22,23,24,25,26,27,28,29,30]. These concepts are illustrated in Figure 1, which summarizes the key cognitive theories that underpin simulation-based learning in surgical education.

Integrating simulations into training programs provides a flexible alternative to the classic “see one, do one, teach one” approach [31]. As Ahmed et al. highlight, moving towards evidence-based simulation should be supported by thorough validation, fidelity testing, and seamless integration into broader cognitive and clinical learning plans [15]. A summary of the most influential cognitive learning theories relevant to simulation-based training is presented in Table 1.

## 4. Insights from Other Surgical Fields

An increasing number of randomized controlled trials show that AI-powered simulators effectively improve surgical education. The following section highlights evidence from research studies in general surgery, neurosurgery, and orthopedics. A comparative overview of key studies from general, neurosurgical, orthopedic, and vascular surgery is provided in Supplementary Table S1.

### 4.1. General Surgery

VR simulation has been shown to improve both technical and non-technical skills in general surgery. In a randomized controlled trial by Palter et al. (2013), virtual reality training was incorporated into a Structured Training and Assessment Curriculum (STAC) and compared to conventional training. Residents in the STAC group outperformed their peers on the first four laparoscopic cholecystectomies (*p* = 0.004 to 0.036) and demonstrated significantly higher non-technical skill scores (*p* = 0.027) [32].

Similarly, Seymour et al. (2002) found that VR-trained residents made significantly fewer intraoperative errors than those trained by traditional methods. Non-VR-trained residents were nine times more likely to temporarily fail to progress (*p* < 0.007) and five times more likely to injure the gallbladder or burn non-target tissue (*p* < 0.04). Overall, the VR group made six times fewer errors per case (1.19 vs. 7.38; *p* < 0.008) [31].

### 4.2. Neurosurgery

Mirchi et al. (2020) introduced the Virtual Operative Assistant (VOA), an AI-powered platform for brain tumor resection training. VOA achieved 92% accuracy in distinguishing experts from novices and provided immediate, benchmark-based feedback to support learning [33].

Yilmaz et al. (2024) demonstrated that real-time AI feedback during simulated neurosurgical tasks significantly improved performance metrics compared to both traditional face-to-face instruction and no-feedback controls [34].

Fazlollahi et al. (2023) further showed that AI feedback improved safety metrics by enhancing bimanual coordination and reducing resection of healthy tissue. However, the study also noted unintended behavioral adaptations, such as slower resection rates and reduced dominant hand speed, emphasizing the need to monitor the cognitive impact of AI on surgical decision-making [35].

### 4.3. Orthopedic Surgery

Lohre et al. (2020) conducted an RCT evaluating immersive VR training in shoulder arthroscopy. VR-trained residents significantly outperformed controls in task efficiency and technical skill. Importantly, VR performance correlated strongly with intraoperative outcomes, supporting the transfer of meaningful skills [36].

Logishetty et al. (2020) examined visuospatial skill acquisition during total hip arthroplasty training in VR. Residents demonstrated a clear learning curve, reaching performance plateaus after ~4 h and achieving expert-level performance in 9 out of 10 metrics. Simulation reduced procedural errors by 79%, operative time by 28%, and hand movement by over 35%. Notably, residents who excelled in VR were also the most accurate in cadaveric assessments, underscoring the predictive value of VR proficiency for real-world performance [37].

### 4.4. Systematic Reviews of Immersive VR in Surgical Training

A systematic review by Mao et al. (2021) examined 17 studies involving 307 participants from four disciplines. Participants trained with immersive VR completed procedures 18% to 43% faster than control groups (*p* < 0.0001). VR trainees also showed better post-intervention checklist scores and more precise implant placement. Importantly, user satisfaction with immersive VR was high, and reports of discomfort were minimal [6].

Sheik-Ali et al. (2019) conducted a narrative review on integrating “Phase 2” VR/AR technologies, which include devices with high-resolution screens and mobile GPUs, into surgical training. In 11 studies, VR/AR methods were consistently linked to faster skill acquisition, better multitasking, higher accuracy, and improved bimanual coordination. However, the authors highlighted significant differences in study designs and stressed the need for standardization and longer-term research [38].

Similarly, Woodall et al. (2023) conducted a comprehensive systematic review analyzing 32 studies that compared extended XR with traditional training methods. The majority of these studies showed enhancements in procedural skills, learner satisfaction, and objective technical scores. Nonetheless, few examined patient-level outcomes (Kirkpatrick level IV), and XR did not significantly surpass standard approaches in knowledge assessments. The authors called for longitudinal research to evaluate clinical translation and cost-effectiveness [39].

Mergen et al. (2024) conducted a scoping review of 69 studies examining VR in medical education, mainly focusing on surgical and emergency medicine. The review found positive outcomes such as shorter procedure times, better skill development, and increased learner engagement. However, it also highlighted significant barriers: high costs, technical challenges, and the absence of standardized evaluation methods. The authors emphasized the need for interdisciplinary collaboration and more defined strategies for integrating VR into curricula [40].

Nassar et al. (2021) emphasized the cognitive basis of VR-based surgical training. Their review pointed out that VR tool effectiveness depends not only on realism and interactivity but also on adherence to established motor learning and deliberate practice theories. Incorporating feedback systems and structured repetition into simulations is identified as vital for sustaining long-term skill retention [41].

## 5. VR Training in Vascular Surgery

VR simulation is recognized as a vital tool in vascular surgery education, allowing trainees to develop and evaluate their technical skills without risking patient safety. The upcoming section summarizes current evidence on VR-based training in vascular surgery, organized by procedure type.

### 5.1. Fundamental Endovascular Skills: Early Simulation and Structured Curricula

Training beginners in endovascular surgery presents unique challenges due to the steep learning curve and limited early patient exposure. Several studies have examined how simulation-based education can bridge this gap. A randomized study involving 50 medical students with no prior endovascular experience compared three learning methods: video instruction, gesture-based simulation, and hands-on training with physical endovascular tools. While students using physical tools reported higher confidence and greater interest in vascular surgery, objective performance improvements were minimal. Only two metrics, guidewire selection and initial vessel positioning, showed modest gains (*p* = 0.045 and *p* = 0.05). This highlights an important caveat: while early simulation boosts engagement and motivation, it may not translate into measurable technical proficiency [42].

To address this, structured simulation curricula have been developed to provide a more comprehensive and skill-focused learning path. One prominent example is the PROSPECT program, which combines e-learning with proficiency-based VR training. In a multicenter evaluation, participants showed significant improvement in technical skills, as indicated by a median Global Rating Scale score increase from 27 to 49 (*p* = 0.027). However, implementation faced logistical barriers, including a lack of protected training time and technical support, leading to high dropout rates. Despite this, over 80% of trainees supported making such training mandatory before real-life procedures [43].

Innovative VR systems with haptic feedback offer another dimension of skill development. Wang et al. developed a simulator that integrated tactile resistance via magnetorheological fluids with real-time visual alerts. After five training sessions, participants improved both safety and speed, spending 15.9% more time in designated “safe zones” and reducing procedure time by nearly 19 s [44]. This suggests that multi-sensory feedback can enhance hand–eye coordination and procedural awareness.

Together, these findings underline the importance of well-structured, multimodal simulation training in developing fundamental endovascular skills. However, confidence gains alone should not be mistaken for competence; curricula must be rigorously designed, supported, and assessed to ensure genuine skill acquisition.

### 5.2. Carotid Artery Stenting

Carotid artery stenting (CAS) is a technically demanding procedure with significant risks, making structured preclinical training essential. In a landmark prospective, blinded, randomized study, Cates et al. evaluated the effectiveness of VR simulation in training experienced interventional cardiologists who were performing carotid angiography for the first time. Participants trained on a high-fidelity endovascular simulator (VIST) demonstrated significantly better performance than those who received traditional, mentored, in vivo instruction. Specifically, the VR-trained group achieved key proficiency benchmarks before treating live patients and exhibited 49% fewer intraoperative errors, 17% shorter procedure duration, and 21% reduction in fluoroscopy time. These results provide compelling evidence for the transferability of skills from VR training to clinical performance. Notably, the study illustrates that simulation is not only effective for novices but also improves procedural safety and efficiency among seasoned operators entering new procedural domains [9].

### 5.3. Aneurysm Repair

Training for endovascular aneurysm repair (EVAR) has historically lacked standardized, proficiency-based simulation curricula, a gap that Moglia et al. sought to address through a pivotal multicenter study using the Angio Mentor VR simulator (Simbionix). Twelve expert vascular surgeons from Italian teaching hospitals, each with experience in over 150 real-life EVAR procedures, were recruited to perform three progressively complex simulated cases. Objective performance metrics, including total procedure time, contrast volume, fluoroscopy duration, and time to contralateral gate cannulation, were used to define benchmark proficiency. This benchmark was set at one standard deviation below the expert average, providing a robust, quantifiable threshold for trainee assessment. Their methodology is based on the Dreyfus model of skill acquisition, which goes beyond just counting case volume. Instead, they focus on competency-based evaluation to better understand and support trainee development. The benchmarks set serve as a clear and consistent way to monitor progress and customize feedback, helping trainees grow more effectively.

Notably, the authors advocate for incorporating these metrics into national or European certification frameworks such as the EBSQ-VASC, pending validation through larger, prospective studies. This research lays the groundwork for a structured, outcome-oriented EVAR training pathway, shifting the paradigm from “time served” to skills demonstrated [45].

### 5.4. Ruptured Abdominal Aortic Aneurysm Management

Managing ruptured abdominal aortic aneurysms (rAAA) demands both rapid technical execution and coordinated team-based decision-making. To address this, Rudarakanchana et al. evaluated the use of immersive VR simulation for training and assessing both technical and non-technical skills in rEVAR [46].

The study employed the VIST-C simulator embedded within a high-fidelity simulated angiosuite environment (ORCAMP). Multidisciplinary teams led by either experienced or trainee endovascular specialists performed rEVAR procedures under realistic emergency conditions. The simulator successfully differentiated performance between experience levels. Experienced leaders achieved proximal control faster (352 vs. 501 s; *p* = 0.047), they used less fluoroscopy time (*p* = 0.016), and only experienced teams completed the whole procedure within the allocated time. In addition to technical metrics, the simulation was highly rated for its realism and educational value, particularly in improving communication, teamwork, situational awareness, and patient safety, all crucial elements in high-stakes vascular emergencies. This study highlights the unique value of immersive VR not only in refining procedural skills but also in fostering critical human factor training. Its integration into vascular education may be especially beneficial for preparing teams to manage rare but life-threatening scenarios, such as rAAA.

### 5.5. Peripheral Arterial Disease

In a landmark study, Van Herzeele et al. demonstrated that combining cognitive pre-training with VR simulation significantly improved procedural outcomes in novice trainees performing iliac angioplasty [7]. Trainees who underwent cognitive preparation achieved stent placement accuracy and residual stenosis rates comparable to those of experienced vascular surgeons. This suggests that mental rehearsal and anatomical understanding are critical adjuncts to technical skills in peripheral arterial disease procedures. These results align with the broader conclusions of Ahmed et al., whose systematic review emphasized the use of VR simulation as an effective tool for teaching, assessing, and facilitating skill transfer in angioplasty and stenting [15]. More recently, Ohashi Torres et al. evaluated the educational impact of VR simulation for lower limb angioplasty among vascular surgery residents [47]. Trainees reported high satisfaction and demonstrated measurable improvements in procedural time, complication handling, and overall technical performance. The study supports the integration of VR modules into PAD-focused curricula, highlighting benefits in both technical proficiency and decision-making under pressure.

Together, these studies reinforce the growing consensus that VR-based simulation is a valuable, evidence-based method for preparing trainees to manage complex PAD interventions with greater precision and safety.

## 6. Other Applications in Vascular Surgery

While VR has proven its value in procedure-specific training, recent advancements extend its role far beyond basic simulation. From AI-assisted performance assessment to real-time haptics, patient-specific rehearsal, and remote coaching, emerging technologies are rapidly reshaping the landscape of vascular surgery education.

### 6.1. AI as an Objective Evaluator

A promising trend is the integration of AI into VR systems to automate skill assessment. Saricilar et al. showed that AI can be used within a high-fidelity simulator to assess procedural skills without relying on human examiners [10]. Similarly, the Fundamentals of Endovascular Surgery (FEVS) model, developed by Duran et al., employed both silicone-based and VR simulation, incorporating motion tracking and error metrics. The study successfully differentiated between competent and non-competent operators (*p* < 0.0001), highlighting AI’s potential to support standardized, proficiency-based certification [48].

### 6.2. Technical Novelty

To improve how realistic and responsive current endovascular simulators are, Li et al. (2024) developed a personalized virtual reality training system for vascular intervention surgery. This system combines Position-Based Dynamics (PBD), Cosserat elastic rods, and quaternion-based shape matching within a unified particle framework. It enables highly realistic simulation of guidewire bending, twisting, and vessel deformation. Users experience smooth, real-time interactions thanks to optimized multi-level continuous collision detection with spatial hashing, achieving an average system latency of 15.54 ms and frame rates up to 64 frames per second. The simulation can handle complex, patient-specific cases, like guiding a wire through bifurcating vessels, all while keeping physical accuracy. Experimental results have shown that the model effectively mimics real-world deformations and procedural behaviors, representing a major step forward in immersive and precise endovascular training [49].

### 6.3. Patient-Specific Rehearsal

Emerging visualization technologies such as AR are gaining traction not only in diagnosis and treatment planning but also as potential tools for surgical training and education. When combined with AI, AR can provide highly intuitive and immersive representations of complex vascular pathologies, which are useful for both clinicians and patients.

A notable example of this approach is a system developed by Lareyre et al. which automatically processes Computed Tomography (CT) scans of patients with AAA, segments the vasculature, and predicts the distribution of wall stress. These data are then rendered as color-coded maps through HoloLens 2 AR glasses. Although initially designed as a patient education tool, the system’s capability to visualize biomechanical stress patterns in real-time 3D has clear potential for integration into personalized preoperative training and PsR in vascular surgery. By simulating anatomical and mechanical complexity specific to the individual, such platforms could improve preparedness for high-risk procedures and enhance operative outcomes [50].

## 7. Official Guidelines on VR-Based Vascular Surgical Education

A recent Europe-wide initiative emphasized the importance of equal access to high-quality simulation-based education (SBE) facilities across the continent. As part of this effort, a consensus-driven prioritization identified 30 technical procedures for inclusion in vascular simulation curricula. The top five prioritized procedures focused on fundamental skills, including basic open and endovascular techniques, vascular imaging interpretation, femoral endarterectomy, and open peripheral bypass. In contrast, 26 procedures (such as peripheral pressure measurement, wound management, open complication management, major amputations, and highly advanced endovascular techniques) were excluded. This ESVS-supported prioritization offers a structured framework for the development of future SBE programs tailored to the evolving needs of vascular surgeons across Europe. Realistic simulation, whether through virtual reality, cadaver courses, or structured programs, was recognized as an essential addition to clinical training, particularly in light of evolving technologies and restricted working hours. Importantly, simulation is not intended to replace hands-on experience, but to enhance learning and increase surgical readiness. This drive to harmonize simulation-based curricula underlines the collective responsibility within the vascular community to adopt modern training strategies that support safer and more effective care for patients across Europe [51].

A joint consensus statement from the Society for Vascular Surgery, the American College of Cardiology, and the Society for Vascular Medicine and Biology recommended the integration of simulation-based training into vascular education. The authors emphasized that simulations with objective performance metrics can help standardize the skills of trainees from different backgrounds and provide realistic and structured opportunities for skill acquisition [52].

## 8. Challenges, Limitations, and Cost-Effectiveness: Future Directions

Despite promising advances, the integration of AI into training and decision-making in vascular surgery presents significant ethical, technical, and economic challenges that must be systematically addressed.

A primary concern is data protection and data security. AI algorithms rely heavily on large clinical, imaging, and, increasingly, genomic datasets. Even when anonymized, this data can potentially be re-identified, especially when matched against other sources. This raises concerns about confidentiality and the ethical use of patient data when training. Another critical point is the opacity of algorithms and their impact on informed consent. Many AI systems operate like “black boxes,” offering little transparency regarding their internal logic or decision-making processes. This lack of explainability can undermine both clinician confidence and patient autonomy, particularly when AI influences treatment decisions or surgical risk stratification. In such contexts, it becomes difficult to obtain genuinely informed consent. The growing threat of cyberattacks in healthcare underscores the need to work with cybersecurity experts to protect sensitive data and maintain the integrity of clinical infrastructures [3].

The clinical validity of AI remains a problem. Without rigorous external validation, overfitting and poor generalizability to broader, more diverse populations are likely, thereby limiting the practical applicability of these models.

Moreover, the issue of algorithmic bias cannot be overlooked. If AI tools are trained predominantly on data from specific demographic groups, there is a tangible risk of performance degradation or harm to underrepresented populations. Rather than bridging health disparities, such tools could inadvertently reinforce them. Addressing this requires the deliberate inclusion of diverse datasets and the implementation of continuous fairness audits during model development and deployment [53,54].

From an educational perspective, emerging AI-driven platforms provide scalable and cost-effective alternatives to traditional instruction. However, the initial investment in high-fidelity simulators, data infrastructure, and interdisciplinary development teams (including engineers, ethicists, and educators) can be substantial. Cost-effectiveness analyses are still limited, and future studies should investigate not only direct economic outcomes but also long-term impacts on competency, complication rates, and the efficiency of the healthcare system. Notably, recent international consensus guidelines have outlined key principles for the ethical implementation of AI in surgical training. These include mandates for data transparency, protection of learner and patient privacy, and proactive strategies to mitigate algorithmic bias [55]. In addition to the previously discussed concerns, it is essential to recognize the significant practical challenges associated with implementing AI- and XR-based surgical training in low-resource settings. Limited access to high-fidelity simulators, inadequate technical infrastructure, and difficulties related to hardware and software maintenance can substantially hinder adoption. Furthermore, a lack of trained faculty or simulation specialists may prevent meaningful integration of these tools into formal educational programs. To address these barriers, scalable and cost-effective alternatives are emerging, such as mobile-based simulators, cloud-based platforms, and collaborative use of simulation resources across institutions. These strategies may help democratize access to advanced training technologies and promote broader implementation, especially in underfunded healthcare systems.

In conclusion, while AI and simulation-based education hold great potential to transform vascular surgery training, their integration must proceed with caution. A balanced approach, prioritizing transparency, validation, and equity, will be essential to realizing their benefits without compromising patient safety or professional integrity. The convergence of predictive analytics, ethical frameworks, and high-fidelity training tools represents a critical opportunity to address longstanding limitations in clinical education and promote equitable, high-quality care in the vascular field [56].

## 9. Conclusions

Virtual reality and other simulation-based techniques are transforming surgical education. Current evidence suggests that VR can accelerate skill development, enhance technical abilities, and boost trainee confidence. Although these benefits are encouraging, the field still needs large-scale, prospective research. Besides the clear advantages, this innovative training approach encourages us to imagine a more structured learning pathway for future vascular surgeons, aimed at improving safety and education at every stage. A well-structured training plan might include the following: Virtual reality training for cognitive skills and basic psychomotor tasks → Simulator-based practice for repeating procedures and receiving feedback → Exposure to animal or cadaver labs to enhance tissue handling skills → Patient-specific rehearsals to understand anatomy and procedural steps → Supervised real-world surgeries, gradually moving toward independence. This multi-layered, adaptable model could offer a more efficient, evidence-based, and patient-safe pathway to surgical mastery, particularly in complex fields such as vascular surgery.

## Figures and Tables

**Table 1:** Cognitive theories.

Theory	Core Idea	What to Do in VR
Kolb’s Learning Cycle [15,22]	Learning happens in four stages: do, reflect, learn, retry	Use real cases, give time to reflect, and repeat practice
Situated Learning [7,23]	People learn best in real-life, relevant situations	Use realistic VR scenarios with patient-specific cases
Cognitive Load Theory (CLT) [24,25]	The brain has limits (too much information slows learning)	Simplify tasks, give feedback, and avoid unnecessary visual clutter
Deliberate Practice/Schema [15,27]	Practice with goals and variation builds flexible skills	Offer feedback-rich, varied VR cases that build motor memory
Fitts–Posner Model [27,28]	Skills go from slow to automatic through stages	Provide repeated, structured practice to reach the “automatic” stage
Mastery Learning [29]	Everyone can master skills with time, feedback, and support	Let learners train at their own pace until they reach a set standard

## Data Availability

No new data were created or analyzed in this study.

## Supplementary Materials

The following supporting information can be downloaded at: https://www.mdpi.com/article/10.3390/medsci13030126/s1. Table S1. Summary of key studies on virtual reality (VR), augmented reality (AR), extended reality (XR), and artificial intelligence (AI) in surgical training across multiple specialties, with a focus on vascular surgery. The table outlines study characteristics, surgical field, technologies used, targeted skills, main findings, and relevance to vascular surgery practice.
