# Supplementary material for: Artificial Intelligence and Extended Reality in the Training of Vascular Surgeons: A Narrative Review
Source: Med Sci (Basel). 2025 Aug 12;13(3):126. doi: 10.3390/medsci13030126 (PMC12372134; doi:10.3390/medsci13030126)
Supplement: Supplementary file 1 [file medsci-13-00126-s001.zip › medsci-3805628-supplementary.pdf]

medical sciences

MDPI

Supplementary Table 1. Summary of key studies on virtual reality (VR), augmented reality (AR), extended reality (XR), and artificial intelligence (AI) in surgical training across multiple specialties, with a focus on vascular surgery. The table outlines study characteristics, surgical field, technologies used, targeted skills, main findings, and relevance to vascular surgery practice.

Study	n	type of study	Surgical Field	Technology Used	Target Skills	Key Findings	Relevance to Vascular Surgery
Palter et al. (2013)	20	randomized single-blinded prospective trial	General Surgery	mixed with addition of VR	Technical + non-technical	VR-based curriculum improved laparoscopic performance and non-technical skills	Supports curriculum-based skill transfer to vascular training
Seymour et al. (2002)	16	prospective, stratified randomized controlled trial	General Surgery	VR	Error prevention	VR group made 6x fewer errors, faster procedure time	Highlights role of VR in patient safety training
Mirchi et al. (2020)	50	Prospective, simulation-based validation study	Neurosurgery	AI + VR (Virtual Operative Assistant)	Benchmarking, skill classification	92% accuracy distinguishing experts from novices	AI-based evaluation transferable to vascular simulation
Yilmaz et al. (2024)	97	RCT	Neurosurgery	Real-time AI	Technical performance	AI feedback improved performance vs. face-to-face instruction	Validates real-time AI feedback for surgical education

Fazlollahi et al. (2023)	60	cohort study	Neurosurgery	AI + VR	Safety, coordination	Improved metrics, unintended cognitive effects observed	Highlights need for careful AI integration in vascular surgery
Lohre et al. (2020)	18	block randomized, intervention-controlled clinical trial	Orthopedics	Immersive VR	Efficiency, psychomotor	Significant improvement in OR metrics	Supports VR as predictor of clinical performance
Logishetty et al. (2020)	32 orthopedic residents plus 4 expert hip surgeons	prospective training study	Orthopedics	VR	Visuospatial skill	79% error reduction, learning curve visualized	Encourages VR use for catheter-based skills training
Mao et al. (2021)	307	systematic review that included 17 primary studies	Mixed surgical fields	Immersive VR	Time, precision	VR reduced task time (18–43%), improved checklist scores	Quantifies broad VR benefits across fields
Sheik-Ali et al. (2019)	Not reported	narrative literature review	Mixed surgical fields	VR/AR	Skill acquisition	Improved multitasking and bimanual dexterity	Stresses need for standardization in vascular simulation

Woodall et al. (2023)	Not reported	Systematic review with 32 studies included (18 randomized controlled trials, 7 comparative studies, 7 systematic reviews)	Mixed surgical fields	XR	Procedural skills	Improved procedural scores, little impact on knowled ge	XR shows promise, but clinical impact needs study
Mergen et al. (2024)	Not applicable	Scoping review protocol	Medical Education	VR	Adoption, infrastructure	Positive outcomes, cost and access barriers highlighted	Reinforces real-world limitations in implementation
Aeckersberg et al. (2019)	50	Randomized controlled trial (three-arm)	Vascular Surgery	Low-fidelity VR	Basic endovascular	Comparable to other training methods	Supports use of accessible VR models
Wang et al. (2017)	Not reported	Non-randomized pilot evaluation	Vascular Surgery	VR + force feedback	Catheter handling	Improved hand–eye coordination and speed	Highlights benefit of haptic feedback
Soenens et al. (2021)	48 starting trainees, of whom 17 completed the programme (65% dropout rate)	multicentre prospective registry stud	Vascular Surgery	VR (PROSPECT)	Stepwise curriculum	Improved skills, but logistical barriers exist	Confirms value of structured vascular VR programs

Cates et al. (2016)	12	prospective, randomised, blinded trial	Vascular Surgery	VR (Carotid stenting)	Technical errors	49% fewer errors, 21% less fluoroscopy	Validated transfer to clinical carotid procedures
Moglia et al. (2020)	Not reported	prospective proficiency definition study	Vascular Surgery	VR	Proficiency benchmarking	Defined objective metrics for EVAR performance	Supports certification-based training models
Rudarakanchana et al. (2014)	22	prospective simulation-based comparative study	Vascular Surgery	Immersive VR (rEVAR)	Team performance	Only experienced teams completed full task	Highlights VR use for emergency team-based training
Van Herzeele et al. (2008)	47	experimental comparative study	Vascular Surgery	VR	Cognitive outcomes	Improved performance in angioplasty tasks	Shows cognitive simulation benefits for endovascular procedures
Ahmed et al. (2010)	Non applicable	review article	Vascular Surgery	VR	Assessment, education	Improved training outcomes for endovascular procedures	One of the first validations of VR in vascular education
